# Sex-specific differences in the growth and population characteristics of Sand crab *Ovalipes punctatus* (De Haan, 1833) in coastal waters of Korea

**DOI:** 10.1038/s41598-024-70959-6

**Published:** 2024-08-29

**Authors:** Hyeon Gyu Lee, Jae Mook Jeong, Youn Hee Choi

**Affiliations:** 1 Fisheries Resources Research Center, National Institute of Fisheries Science, Tongyeong, 56034 Korea; 2 Division of Fisheries Life Sciences, Pukyong National University, Busan, 48513 Korea

**Keywords:** Sand crab, *Ovalipes punctatus*, Korea, Allometric growth, Sexual dimorphism, Von Bertalanffy growth curves, Community ecology, Population dynamics, Marine biology

## Abstract

The sex-specific differences in the growth and population characteristics of the high-commercial-value sand crab *Ovalipes punctatus* were investigated in Korea. The estimated allometric growth between the sexes showed significant differences in all morphometric measurements. In the classification of growth types, carapace width-chela length exhibited positive and negative allometric growth in males and females, respectively. Carapace width-abdominal width showed positive relative growth in both sexes, and orbital spine width exhibited negative relative growth in both sexes. Consequently, sexual dimorphism was evident in all measured traits. Growth parameters estimated using the ELEFAN function of the FiSAT II program indicated higher values in males compared to females. Asymptotic length (CW_∞_) for males was estimated at 139.2 mm, whereas for females it was 116.6 mm. Additionally, the growth coefficient (K) was higher in males (0.65) than in females (0.54), suggesting faster growth in males. The winter point (WP) was 1 for males and 0.7 for females, indicating slower growth in males during the colder December and slower growth in females during the spawning period in August. The modified von Bertalanffy growth curves indicated asymptotic growth in all sexes, and the growth performance index (φ') showed higher values in males (4.10) compared to females (3.87), reflecting differences in growth curves. The steady increase in recruitment rates from July to September was associated with the appearance of larvae and their subsequent growth into juveniles, leading to their recruitment into the population during this period. Therefore, *O. punctatus* exhibited sex-specific differences in growth parameters, suggesting distinct growth strategies between the sexes.

## Introduction

Crabs have various life history strategies, such as complex growth patterns because of molting and the meroplanktonic phase^1^, the lack of direct age-estimating traits, and growth variability, which render crab resource management difficult^2^. However, because crabs with hard exoskeletons barely undergo morphological changes owing to short-term external influences, more accurate morphological measurements are possible than that with vertebrates and can be useful in identifying the morphological characteristics of crabs and studying populations^3^.

In the study of crabs, length–frequency data are used as key data for the life history of the species, resource management, and development of fishery and aquaculture technology^4^. Morphological changes are used to determine sexual maturity and study allometric growth changes between the changed part and other parts^5^. These differences in phenotypes (such as sexual dimorphism) markedly impact the commercial value of crab species^6–8^. Because these studies have broad applicability in explaining the growth patterns of crabs^9^, they are essential for understanding the ecological roles of species in populations and benthic communities^1^.

Research on these crab species primarily focuses on commercial species^10^, with the superfamily Portunoidea being particularly notable for its economic value^11^. In Korea, the main commercially harvested Portunoidea species include the swimming crab *Portunus trituberculatus* (Miers, 1876), the Asian paddle crab *Charybdis japonica* (A. Milne-Edwards, 1861), and the sand crab *Ovalipes punctatus* (De Haan, 1833). Especially for *P. trituberculatus* and *C. japonica*, both basic ecological research and fisheries resource management policies, such as closed seasons and minimum size limits, have been established to prevent resource depletion^12^. However, despite the high consumption of the sand crab *O. punctatus* in Korea, no ecological studies or fisheries resource management policies have been implemented for this species.

*Ovalipes punctatus* is a species well-adapted to a wide range of temperatures and salinities, possessing strong swimming and burrowing abilities^13^. This species inhabits sandy shores in subtropical and temperate regions worldwide^14^ and is found throughout the waters of Korea^15–17^. It is particularly abundant along the East Sea coast, the West Sea, Jeju Island, the Korea-Japan Intermediate Zone, and the East China Sea^17–21^. In Japan, *O. punctatus* is a commercially valuable species sold at relatively high prices^22^, and in China, its catch has been gradually increasing since the 1980s, making it one of the most important commercial crabs^23^. Similarly, in Korea, the demand for *O. punctatus* has surged recently as an alternative to the highly priced swimming crab. However, this increased demand has led to overfishing of *O. punctatus* in areas such as the East China Sea and Yellow Sea, raising concerns about population growth and recruitment failure^24^. Additionally, due to their body size, these crabs are easily captured by fishing gear with large mesh sizes, necessitating regulatory measures for resource protection^25^.

No related studies have been conducted in Korea despite the high consumption of *O. punctatus*. Therefore, this study aimed to analyze ecological characteristics, such as allometric growth and sexual dimorphism between sexes, classify cohorts in the population, and estimate von Bertalanffy growth function (VBGF) and recruitment patterns using morphometric measurement data of *O. punctatus*. The results of this study have implications for resource management and policy establishment regarding sand crabs in Korea.

Therefore, this study aimed to analyze ecological characteristics, such as allometric growth and sexual dimorphism between sexes, classify cohorts in the population, and estimate von Bertalanffy growth function (VBGF) and recruitment patterns using morphometric measurement data of *O. punctatus*. Understanding the growth characteristics of this species, combined with reproductive ecological studies, enables the establishment of more robust and systematic resource management policies, providing a basis for implementing legal regulations such as closed seasons and minimum size limits. The results of this study have implications for resource management and policy establishment regarding sand crabs in Korea, and they will also offer reliable estimates to countries that consume this species, laying the groundwork for sustainable fisheries.

## Materials and methods

### Approval for animal experiments

The *Ovalipes punctatus* used in this study does not require a permit for collection, and it is not currently considered an endangered or protected species in Korea. All experiments were conducted in accordance with the best practices to minimize animal suffering and followed the guidelines established by the Korean Association for Laboratory Animals in the Education on the Use and Management of Laboratory Animals. Also, all animal experiments conducted in this study were in compliance with the ARRIVE guidelines^26^. 

### Sample collection

**Fig. 1:**
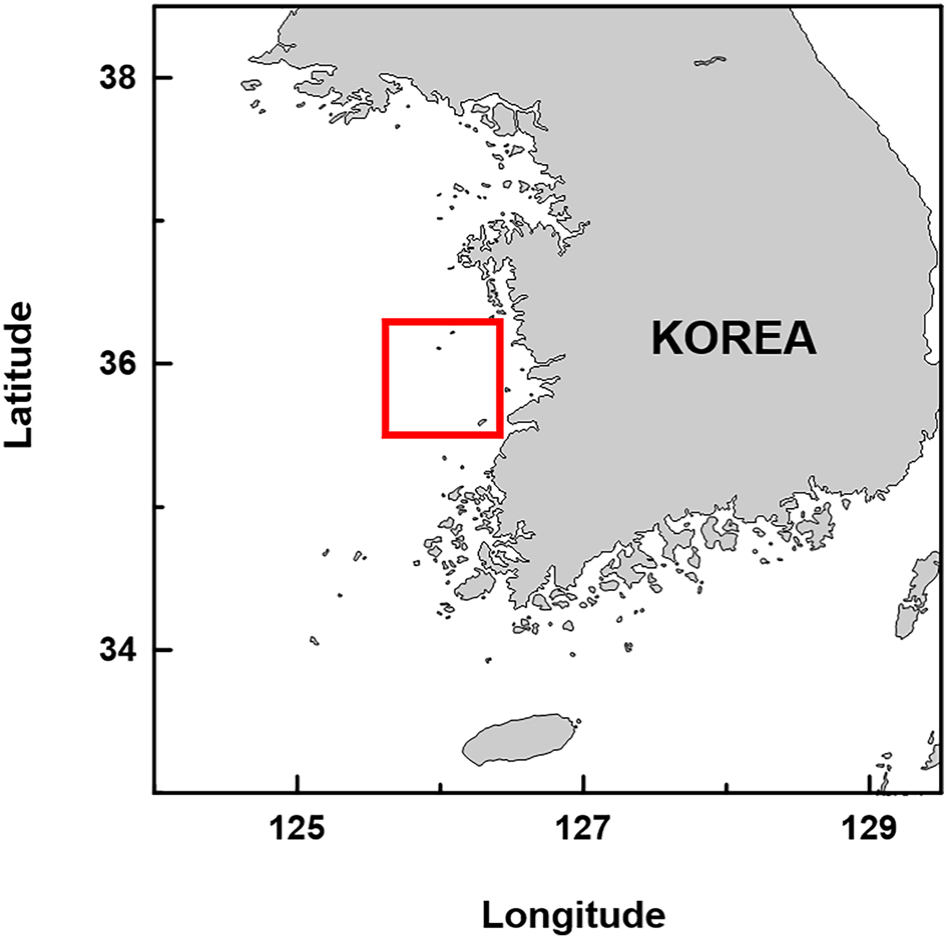
Sampling area of sand crab *Ovalipes punctatus* in coastal waters of Korea in 2021.

The *Ovalipes punctatus* samples were collected from the Yellow Sea of Korea (Coordinates of sampling area: 35° 15′ 00′′ N, 125° 15′ 00′′ E.) between January and December 2021 using the experimental bottom trawl (cod-end mesh size: 20 mm) of the National Institute of Fisheries Science research vessels (Tamgu 20–22) (Fig. 1).

### Morphometric measurements

**Fig. 2:**
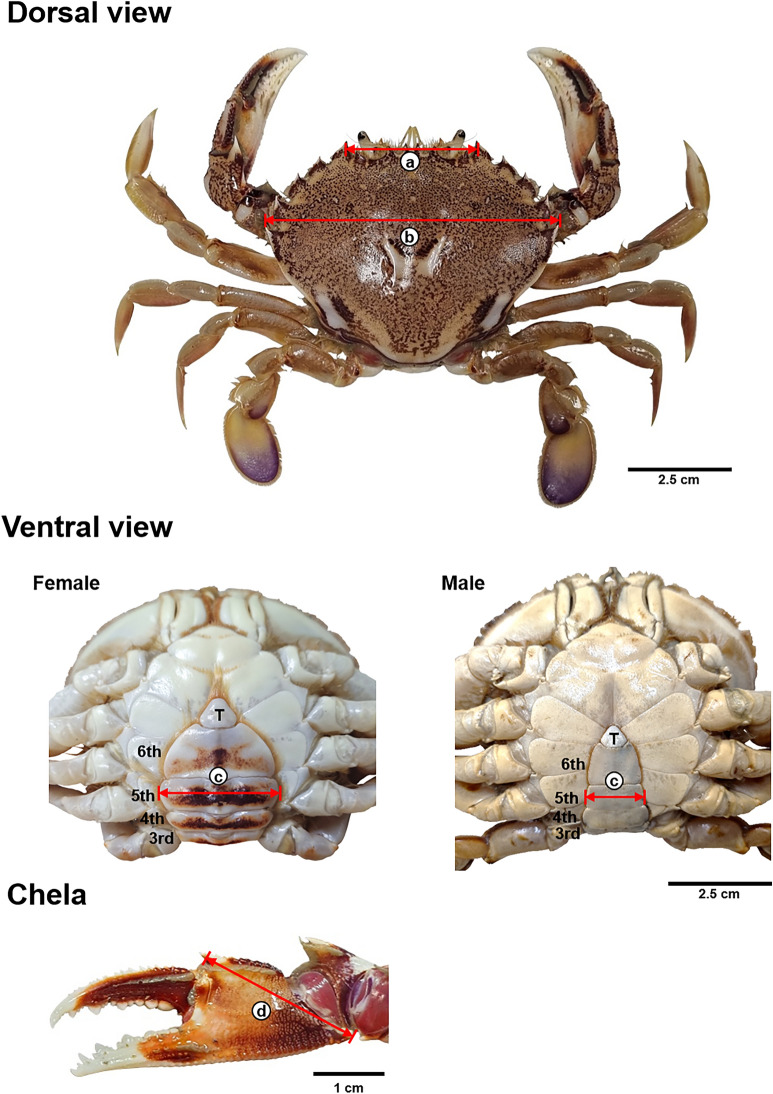
Morphometric measurements of sand crab *Ovalipes punctatus*. (**a**) orbital spine width (OW), (**b**) carapace width (CW), (**c**) abdominal width (AW), and (**d**) chela length (ChL). *T* telson.

For the collected samples (males: 294, females: 183), carapace width, chela length, abdominal width, and orbital spine width were measured to the nearest 0.01 mm using a vernier caliper, and wet weight was measured to a resolution of 0.01 g using an electronic scale (CAS CUW6200H, Korea) (Fig. 2). For individuals with missing limbs when measuring wet weight, errors were minimized by measuring the weight of the intact legs and calculating the potential weight of the missing limbs, referring to the method of Zaleski and Tamone^27^.

### Allometric growth analysis

The following formula was used to estimate the allometric growth for each morphometric measurement:

Allometric growth of carapace width-body weight

1 $$BW={aCW}^{b},$$

where BW is the body weight, CW is the carapace width, and a and b are constants. The constant b is the allometric growth coefficient; b < 3 indicates negative allometric growth (body weight increases slower than body length), b > 3 indicates positive allometric growth (weight increases faster than body length), and b = 3 indicates isometric growth^28^.

Allometric growth of carapace width-chela length

2 $$CW=aChL-b,$$

where CW is the carapace width, ChL is the chela length, and a and b are constants.

Allometric growth of carapace width-orbital spine width

3 $$CW=aOW-b,$$

where CW is the carapace width, OW is the orbital spine width, and a and b are constants.

Allometric growth of carapace width-abdominal width

4 $$CW=aAW-b,$$

where CW is the carapace width, AW is the abdominal width, and a and b are constants.

To classify the type of allometric growth of the morphometric measurement data, excluding weight data, we converted the existing linearized equation into the form Y = aX^b^, where Y is the dependent variable, and X is the independent variable. In the allometric growth analysis of most crabs, the carapace width is used as the independent variable X^29^. The classification of allometric growth types has been previously described^30^. b < 1 indicates negative allometric growth, where Y grows slower than X; b > 1 indicates positive allometric growth, where Y grows faster than X, and b = 1 indicates isometric growth. Sexual dimorphism between the sexes was tested using the Kolmogorov–Smirnov (K-S) test using the length–frequency distribution for each morphometric measurement.

### Analysis of population characteristics

Mode separation of the cohort was performed after analyzing the average carapace width and standard deviation using Bhattacharya’s method, and each mode was accurately represented using Hasselblad’s normal separation (NORMSEP) method^31^. The reliability of the groups was determined using a separation index (S.I.). The recruitment pattern was estimated using the FiSAT II program.

### Analysis of the modified von Bertalanffy growth function

The growth of *O. punctatus* was described using the modified von Bertalanffy growth function (VBGF), which represents the monthly carapace widths of females and males as a length–frequency distribution at 5 mm intervals:

5 $${CW}_{t}={CW}_{\infty }\left[1-{e}^{[-K(t-{t}_{0})-\left(\frac{CK}{2\pi }\right)\text{sin}2\pi (t-{t}_{s})]}\right],$$

where CW_t_ is the carapace width at age t, CW_∞_ is the asymptotic carapace width, K is the intrinsic growth rate, t_0_ is the theoretical age at zero length, C is the amplitude of seasonal growth oscillation, t_s_ is the age at the beginning of growth oscillation, and winter point (WP; t_s_ + 0.5) is the season of year when growth is the slowest. t_0_ was estimated using Veiga’s method^32^; however, it was set to 0 because no results for the CW of the early larvae of *O. punctatus* were obtained*.* These growth parameters were estimated using ELEFAN in the FiSAT II program^33^, a non-parametric estimation method, and the reliability was expressed as the R_n_ value.

The growth performance index between the sexes was analyzed and compared using the following formula^34^:

6 $${\varphi }{\prime}=2{\mathit{log}}_{10}{CW}_{\infty }+{\mathit{log}}_{10}K,$$

where φ' is the growth performance index, CW_∞_ is the asymptotic CW, and K is the intrinsic growth rate.

### Data analysis

Differences in allometric growth between the sexes of *O. punctatus* were tested using analysis of covariance (ANCOVA). In all allometric growth graphs, the independent variable is carapace width, and the dependent variables are chela length, orbital spine width, and abdominal width. Additionally, statistical significance was determined using a t-test for the classification of allometric growth types. Kolmogorov–Smirnov test (K–S test) was performed to the analyze the length frequency distribution of the male and female individuals. Additionally, t-test was used to determine the statistical significance of the classification of allometric growth types, and the Kolmogorov–Smirnov test (K–S test) was performed to analyze the carapace width frequency distribution of males and females. All statistical analyses were performed using Microsoft Excel 2016 (Microsoft Corporation, Redmond, Washington, USA) and PAST 4.03 (PAleontological STatistics version 4.03)^35^.

## Results

### Allometric growth

#### Allometric growth of carapace width-body weight

**Fig. 3:**
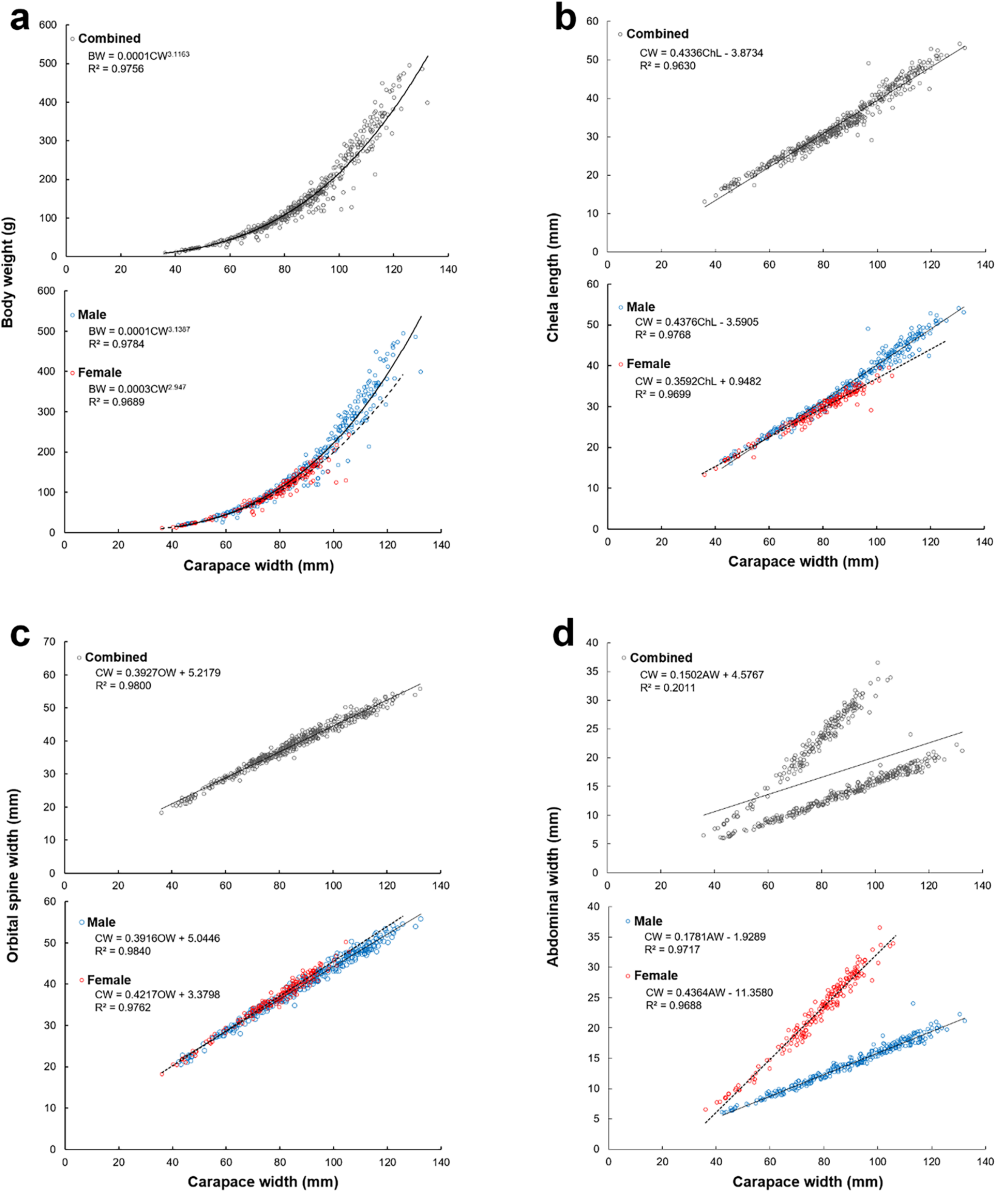
Allometric growth graph of sand crab *Ovalipes punctatus* in the Yellow Sea of Korea in 2021. (**a**) CW–BW, (**b**) CW–ChL, (**c**) CW–OW, and (**d**) CW–AW. *CW* carapace width, *BW* body weight, *ChL* chela length, *AW* abdominal width, *OW* orbital spine width.

The allometric growth between CW and body weight of all individuals (n = 477) was estimated as BW = 0.0001CW^3.1163^ (R^2^ = 0.9756, b > 3, n = 477). Males showed positive allometric growth with BW = 0.0001CW^3.1387^ (R^2^ = 0.9784, b > 3, n = 294), and females showed negative allometric growth with BW = 0.0003CW^2.947^ (R^2^ = 0.9689, b < 3, n = 183) (t-test: p < 0.01) (Fig. 3a). Analyzing the allometric growth of carapace width-body weight between sexes showed a significant difference in the slope (ANCOVA: F = 15.30, p < 0.001).

#### Allometric growth of carapace width-chela length

The allometric growth between CW and chela length of all individuals was estimated as CW = 0.4336ChL − 3.8734 (R^2^ = 0.9630, n = 477); the allometric growth of males and females was CW = 0.4376ChL − 3.5905 (R^2^ = 0.9768, n = 294) and CW = 0.3592ChL + 0.9482 (R^2^ = 0.9699, n = 183), respectively (Fig. 3b). Analyzing the allometric growth of the carapace width-chela length between the sexes showed a significant difference in the slope (ANCOVA: F = 103.328, p < 0.001).

#### Allometric growth of carapace width-orbital spine width

The allometric growth between the carapace width and orbital spine width of all individuals was estimated as CW = 0.3927OW + 5.2179 (R^2^ = 0.9800, n = 477); the allometric growth of males and females was CW = 0.3916OW + 5.0446 (R^2^ = 0.9840, n = 294) and CW = 0.4217OW + 3.3798 (R^2^ = 0.9762, n = 183), respectively (Fig. 3c). An analysis of the allometric growth of the carapace width-orbital spine width between sexes showed a significant difference in the slope (ANCOVA: F = 25.61, p < 0.001).

#### Allometric growth of carapace width-abdominal width

The allometric growth between carapace width and abdominal width of all individuals was estimated as CW = 0.1502AW + 4.5767 (R^2^ = 0.2011, n = 477); the allometric growth of males and females was CW = 0.1781AW − 1.9289 (R^2^ = 0.9717, n = 294) and CW = 0.4364AW − 11.3580 (R^2^ = 0.9688, n = 183), respectively (Fig. 3d). An analysis of the allometric growth of the carapace width-abdominal width between sexes showed a significant difference in the slope (ANCOVA: F = 2626.11, p < 0.001).

### Classification of allometric growth types

The allometric growth types were classified by converting the existing linearized equation into the form Y = aX^b^ (Table 1). The allometric growth types of the carapace width-chela length were ChL = 0.2626CW^1.0914^ (R^2^ = 0.9830, b > 0, n = 294) and ChL = 0.4245CW^0.9693^ (R^2^ = 0.9692, b < 0, n = 183) for males and females, respectively. Males showed positive allometric growth close to isometric growth, whereas females showed relatively negative allometric growth, which was close to isometric growth (t-test: p < 0.01).

**Table 1:** Allometric growth equations and growth type of sand crab *Ovalipes punctatus* in the Yellow Sea of Korea in 2021.

Allometric growth	Male				Female			
	n	Equation	Type	p	n	Equation	Type	p
CW–ChL	294	ChL = 0.2626CW^1.0914^	** + **	p < 0.01	183	ChL = 0.4245CW^0.9693^	–	p < 0.01
CW–AW	294	AW = 0.0788CW^1.1517^	** + **	p < 0.01	183	AW = 0.0178CW^1.6372^	+	p < 0.01
CW–OW	294	OW = 0.8122CW^0.8683^	**–**	p < 0.01	183	OW = 0.7414CW^0.8933^	–	p < 0.01

+ Positive allometric growth, – Negative allometric growth, *CW* carapace width, *ChL* chela length, *AW* abdominal width, *OW* orbital spine width.

The allometric growth types of the carapace width-abdominal width were AW = 0.0788CW^1.1517^ (R^2^ = 0.9819, b > 0, n = 294) and AW = 0.0178CW^1.6372^ (R^2^ = 0.9815, b > 0, n = 183) for males and females, respectively. Males showed positive allometric growth close to homogeneous growth, whereas females showed strong positive allometric growth (t-test: p < 0.01).

The allometric growth types of the carapace width-orbital spine width were OW = 0.8122CW^0.8683^ (R^2^ = 0.9824, b < 0, n = 294) and OW = 0.7414CW^0.8933^ (R^2^ = 0.9869, b < 0, n = 183) for males and females, respectively. Males and females showed negative allometric growth (t-test: p < 0.01).

### Sex-specific carapace width frequency distribution

**Fig. 4:**
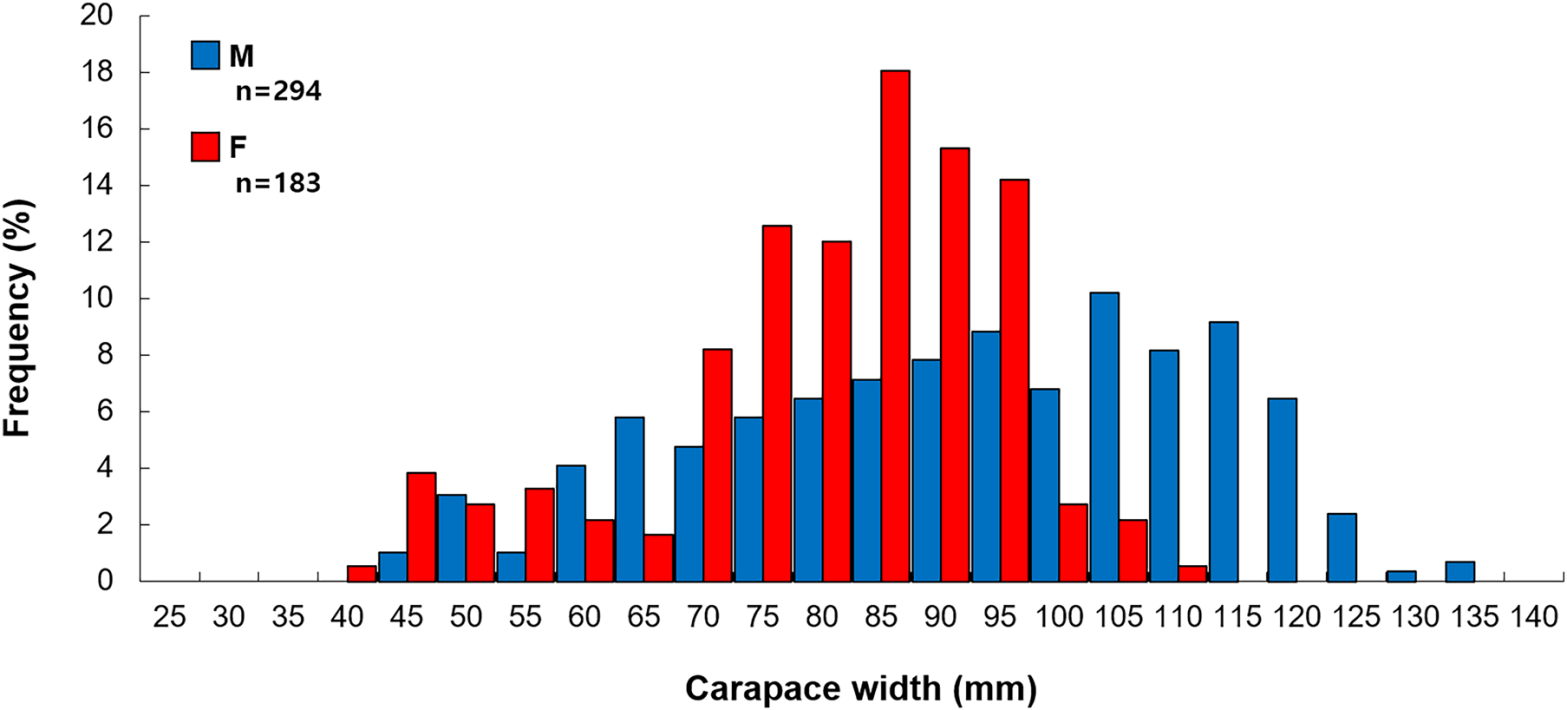
Length frequency distribution of sand crab *Ovalipes punctatus* in the Yellow Sea of Korea in 2021. *M* males, *F* females.

The carapace width frequency distribution of the *O. punctatus* showed a range of 42.3–132.5 mm (mean ± SD: 89.7 ± 20.5 mm) for males and a range of 36.1–105.8 mm (mean ± SD: 78.1 ± 14.0 mm) for females. The K–S test results for the male and female carapace width frequency distributions revealed a significant difference between the sexes (Z = 7.325, p < 0.001) (Fig. 4).

### Population characteristics

#### Mode analysis of Bhattacharya’s method

**Fig. 5:**
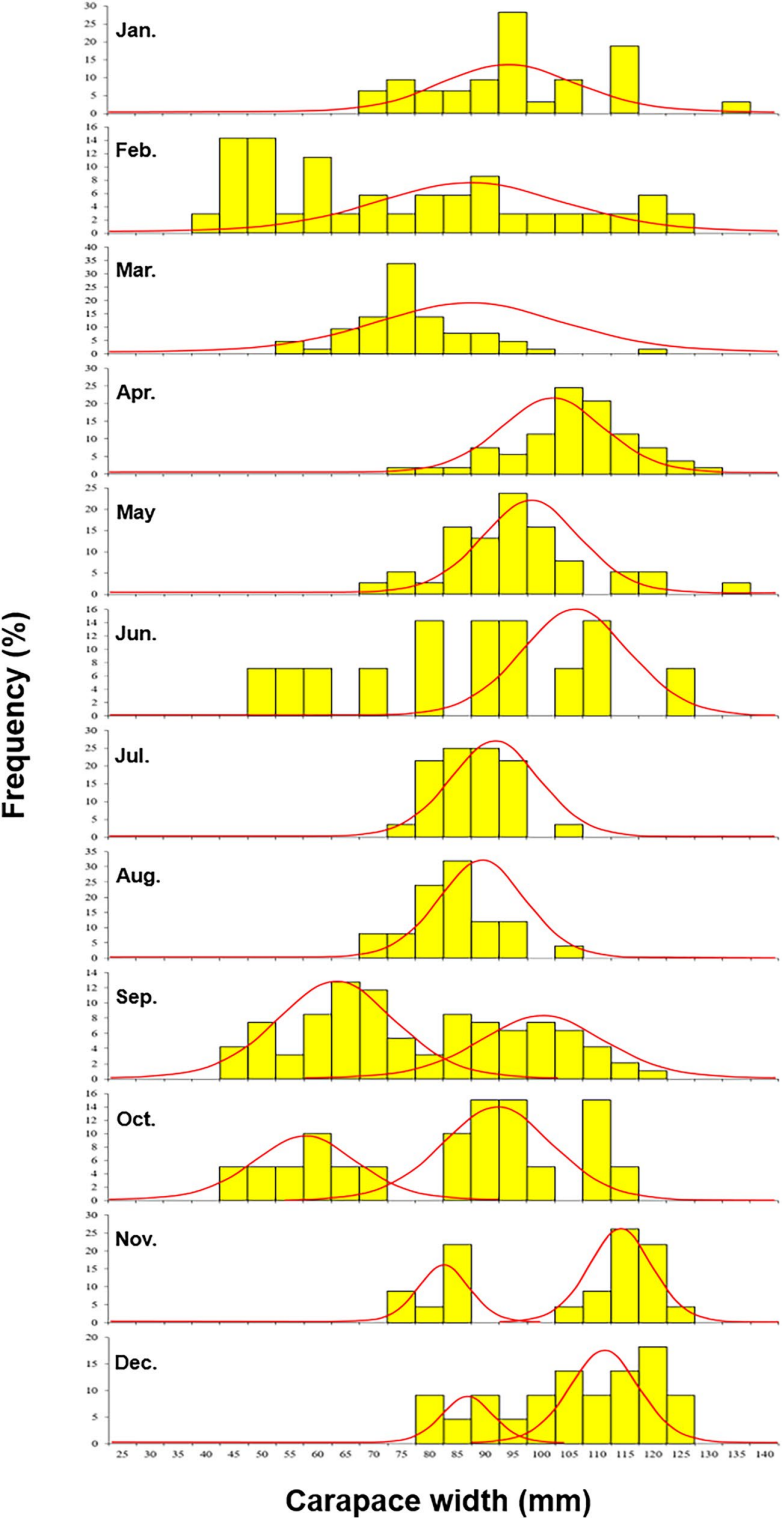
Monthly modes separation of length–frequency distribution and normal distribution curves of sand crab *Ovalipes punctatus* using NORMSEP.

The carapace width of the crabs per month was expressed as a length–frequency distribution, and mode separation was performed using Bhattacharya’s method in the FiSAT II program (Fig. 5). During the research period from September to December, the distribution was separated into two modes, with S.I. values of 3.2, 4.4, 7.4, and 3.7, respectively.

The modes separated in each month were 63.1 ± 10.2 mm and 96.5 ± 10.9 mm in September; 57.6 ± 7.9 mm and 97.4 ± 10.3 mm in October; 81.9 ± 4.3 mm and 116.0 ± 4.9 mm in November; and 86.1 ± 5.5 mm and 112.4 ± 8.9 mm in December.

#### Growth equation estimation using the modified VBGF

**Fig. 6:**
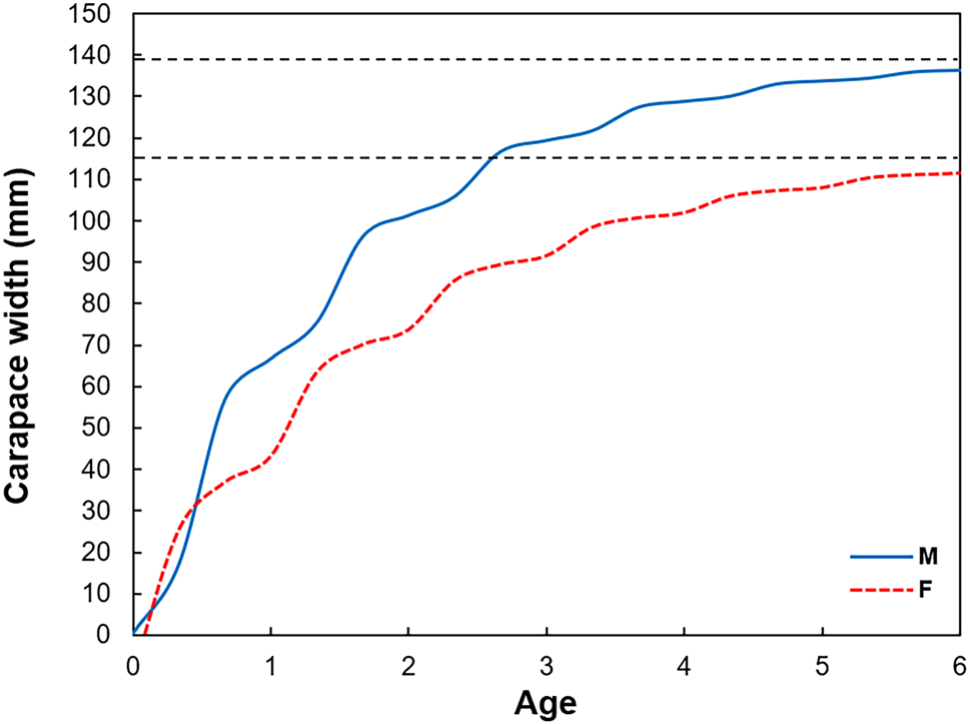
The modified von Bertalanffy growth curves for males and females of sand crab *Ovalipes punctatus* based on length–frequency distributions. CW_∞_ are represented by the black dashed line. *M* males, *F* females.

CW_∞_ was estimated as 139.2 mm and 116.6 mm for males and females, respectively; thus, females were smaller than males (Fig. 6). K was 0.65 year^−1^ for males and 0.54 year^−1^ for females, thereby showing higher values for males. C was 0.1 for both sexes, and WP was 1 and 0.7 for males and females, respectively. Males had the slowest growth in December and females in August. The goodness of fit (R_n_) of all growth parameters was 0.263 and 0.335 for males and females, respectively (Table 2).

**Table 2:** Modified von Bertalanffy growth parameter estimation using ELEFAN analysis of length–frequency data for males and females.

Growth parameters	Sex	
	Male	Female
CW_∞_	139.2	116.6
K	0.65	0.54
t_0_	0	0
C	0.1	0.1
WP	1	0.7
φ’	4.10	3.87
R_n_	0.263	0.335

*CW*_*∞*_ asymptotic length (mm), *K* growth coefficient (yr^−1^), *t*_*0*_ theoretical age at zero length (years), *C* amplitude of growth oscillation, *WP* winter point, *φ'* growth performance index, *R*_*n*_ score function.

$$\text{Male}=139.2\left[1-{e}^{[-0.65(t-0)-\left(\frac{0.1\times 0.65}{2\pi }\right)sin2\pi (t-0.5)]}\right]$$

$$\text{Female}=116.6\left[1-{e}^{[-0.54(t-0)-\left(\frac{0.1\times 0.54}{2\pi }\right)\mathit{sin}2\pi (t-0.2)]}\right]$$

The growth performance index (φ') was 4.10 and 3.87 for males and females, respectively, indicating that males grew faster than females.

At a 95% confidence level, the confidence interval (CI) for males was 18.42, with the upper limit and lower limit being 120.33 mm and 83.49 mm, respectively. For females, the confidence interval (CI) was 15.49, with the upper limit and lower limit being 95.75 mm and 64.76 mm, respectively.

#### Recruitment patterns

**Fig. 7:**
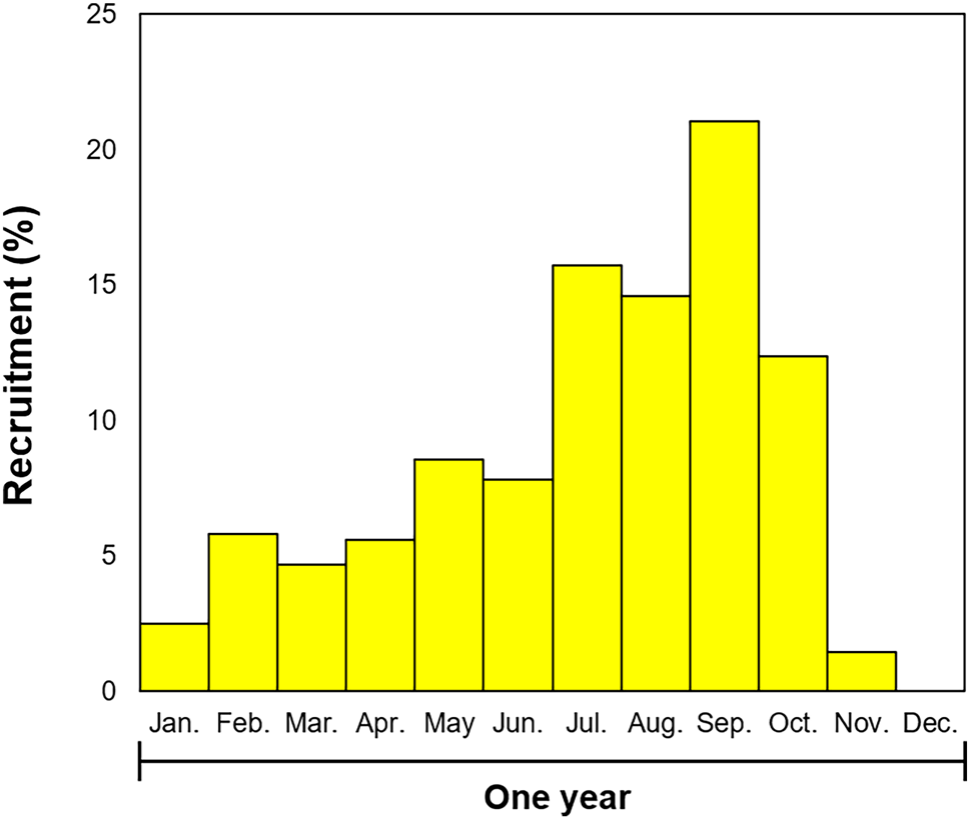
Annual recruitment pattern of sand crab *Ovalipes punctatus* as estimated by ELEFAN II routine.

After estimating the growth parameters (CW_∞_, K, C, and WP) using ELEFAN in the FiSAT II program, monthly recruitment patterns were estimated. The recruitment rate of *O. punctatus* did not exceed 10% from January to June; however, the rate increased to > 15% from July and reached the highest (21.1%) in September. Subsequently, the recruitment rate gradually decreased, showing a rapid decline from November (Fig. 7).

## Discussion

The most frequently used measurement traits in crustacean growth studies are body weight, carapace length, and carapace width^36^. The maximum CW of male and female *O. punctatus* were 115.2 mm and 102.8 mm in Japan^22^, 63.8 mm and 63.2 mm in South Africa^37^, and 98 mm and 82 mm in China^23^, respectively. Sasaki and Kawasaki^22^ reported male and female crabs with maximum CWs of 115.2 mm and 102.8 mm, respectively, whereas Wang et al*.*^23^ reported widths of 98 mm and 82 mm, respectively. In the present study, the maximum CWs of *O. punctatus* in Korea were 132.5 mm and 105.8 mm for males and females, respectively, indicating that all sexes showed larger CWs than those reported in previous studies.

Growth refers to the increase in body volume resulting from the increase in body length and weight, and it can be divided into absolute growth, which represents the growth amount over time, and allometric growth, which describes the growth of other body parts relative to the increase in body length^38^.The allometric growth equation of body length and weight plays an important role in fisheries because it can estimate changes in body weight according to body length and can be used as an indicator of reproduction, feeding, and adaptability to the environment^4,39,40^. Males generally have larger sizes and weigh than females^41^. Mud crab *Scylla olivacea* (Herbst, 1796) (Brachyura: Portunidae) showed positive allometric growth in males and negative allometric growth in females^42^; in snow crab *Chionoecetes opilio* (Fabricius, 1788) (Brachyura: Oregoniidae), males had a higher body weight than did females^43^. *Ovalipes catharus* (White in White & Doubleday, 1843) also exhibited a higher body weight in males compared to females^29^. Based on the carapace width-weight allometric growth results of this study, males exhibited positive allometric growth (b = 3.14), while females showed negative allometric growth (b = 2.95). This difference is attributable to a sex-specific energy allocation strategy for crustaceans, in which males allocate more energy for body growth to gain an advantage in mating competition by increasing their body size, and females have smaller sizes and slower growth because they invest more energy in reproductive processes^5,44^.

In crabs, sexual dimorphism occurs primarily in the chelae, abdomen, and first pleopod, and the size of the specific part increases significantly upon molting^5^. The chelae of crabs are important for reproduction and social behavior^45^. Males have advantages in combat and courtship behavior because of sexual dimorphism in the chela^5^, and the chelae also play an important role in sexual appeal and mating during communication of sexual behavior, such as courtship^46^. In *Johngarthia planata* (Stimpson, 1860) of the family Gecarcinidae, the chelae of males are larger than those of females, and males show positive allometric growth^47^. A study on the allometric growth of *Portunus pelagicus* (Linnaeus, 1758) showed that the chelae of males were larger and showed positive allometric growth^48^. Du Preez and McLachlan^37^ reported that *O. punctatus* males had longer chelae than females. In the present study, a significant difference was observed in the allometric growth of carapace width-chela length between the sexes. For the chela growth type, males showed positive allometric growth (b = 1.09) and females showed negative allometric growth (b = 0.96). Different types of chela growth are related to reproduction and spawning, and the presence of larger chelae in males is advantageous in mating competition^47^.

In the present study, R^2^ was low (0.20) only for the allometric growth of abdominal width combined with both sexes because the abdominal width showed stronger sexual dimorphism than the other measured traits. In previous studies of the genus *Ovalipes*, allometric growth of abdominal width was more common in females than in males, and all females showed strong positive allometric growth^29,37,49^. In the present study, there was a significant difference in the allometric growth of carapace width-abdominal width between the sexes, and sexual dimorphism was observed in the frequency distribution of abdominal width. In addition, for growth type, the b-value of all sexes was greater than 1, indicating positive allometric growth. The b-value of females was 1.63, indicating strong positive allometric growth. Additionally, in the study by Davidson and Marsden^29^, the abdominal growth of female *O. catharus*, a species in the same genus, exhibited two growth phases, primarily occurring around a carapace width of 30 − 40 mm. This phenomenon, often seen in many decapods, is associated with the pubertal molt, which results in a marked increase in the slope of the allometric growth graph. In our study, while the *O. punctatus* did not show a clear separation, there was a slight change in the slope between the 40–50 mm carapace width range on the graph. However, to accurately determine the pubertal molt interval and its relationship with sexual maturity in *O. punctatus*, further collection of smaller specimens and integration with reproductive ecological research is required.

Crabs can be divided into two groups (narrow and wide) based on the distance between the eyes. *O. punctatus* belongs to a group of species with a wide width, and species in this group can recognize the exact distance of an object in a three-dimensional space because their visual fields overlap significantly. This is advantageous for searching for prey and recognizing predators, thereby increasing survival rates^50^. In the present study, there was a significant difference in the allometric growth of the carapace width-orbital spine width between the sexes. Moreover, sexual dimorphism was observed in the frequency distribution of orbital spine width, and the growth type showed negative allometric growth in all sexes (b < 1). This negative allometric growth of the orbital spine width is a phenomenon that also occurs in most crabs. This phenomenon restricts the growth of the anterior part of the carapace such that it grows slower than the rest of the carapace because a blind spot may occur if the gap is extremely wide^51^.

Crustaceans do not exhibit age traits, and their growth has been studied using the length–frequency method^38^. In the NORMSEP results, two modes appeared from September to December, and the S.I. of each mode was > 2, indicating that the age groups were well separated. The high S.I. in the results is considered that the number of modes in the frequency distribution of body length was clearly shown. Because the variance in body length for each age was small, as *O. punctatus* individuals have short lifespans and are fast-growing^52^. However, research on crab growth presents difficulties in interpreting data because collecting juvenile crabs is unfeasible in the early stages of settlement^53^. Therefore, collecting samples according to body length is necessary for the collection of various crab stages to accurately separate the age groups and estimate the lifespan of crabs.

Crustacean growth exhibits an asymptotic pattern owing to molting and seasonal influences^41^. These asymptotic patterns are short and discontinuous because crustaceans grow by molting. The VBGF in this study used a modified growth equation that considered seasonal fluctuation factors and molting, which is different from the existing growth equations, as shown in the resulting graph.

The estimated growth parameters (CW_∞_, K, C, and WP) were 139.2 mm, 0.65 year^−1^, 0.1, and 1 and 116.6 mm, 0.54 year^−1^, 0.1, and 0.7 for males and females, respectively. Thus, the CW_∞_ and K of *O. punctatus* were larger in males than in females^54^, which is the same pattern as the parameter estimation growth results for *Portunus segnis* (Forskål, 1775)^55^, *Portunus trituberculatus* in the East China Sea^56^, and *P. trituberculatus* in the Yellow Sea^57^. The difference in variables between the sexes is assumed to be caused by the differences in reproductive strategies, in which males gain an advantage in mating behavior by focusing on body growth, whereas females use more energy to develop internal reproductive organs. The seasonal growth oscillation (C) estimated by the growth curve was 0.1 for both sexes, indicating that *O. punctatus* showed marginal seasonal oscillation in growth. The period for slowest growth (WP) was December (1) for males and August (0.7) for females. These results are attributable to low water temperature in winter, which affects the growth of males, and August is the spawning season for females. These growth characteristics were also observed in the intertidal mud crab, *Macrophthalmus japonicus* (De Haan, 1835)^58^.

The growth performance index (φ') is useful for comparing growth under various environmental conditions^57^. This index was higher in *O. punctatus* males than in females because females invest more energy in the maturation of gonads than in body growth, and they suppress feeding, which causes low growth rates and prevents or delays molting^59^. Similarly, the finding that males grow faster than females has also been observed in the orange mud crab, *Scylla olivacea*, which belongs to the same superfamily, Portunoidea^42^. However, in the case of the swimming crab, *Portunus trituberculatus*, which also belongs to the same superfamily, females were found to grow faster than males^54^. Such sex-specific growth differences among decapod species are common and have been recorded in various previous studies^59^.

The results indicated that the recruitment pattern of *O. punctatus* aligned with that of *Portunus trituberculatus*, which has a similar spawning season and inhabits the same Yellow Sea^54^. According to Lee et al.^60^, the larvae of *O. punctatus* in Korea appear from April to June, and the larvae released from ovigerous females grow into juveniles approximately 30 days later^61^. The continuous increase in recruitment rates from July to September observed in this study is associated with the appearance of larvae and their subsequent growth into juveniles, leading to their recruitment into the population during this period.

In crustacean studies, estimating growth parameters and applying mathematical models to arrive at von Bertalanffy growth function and population cohorts provides reliable estimates of crustacean species^41^. In addition, studying growth and population characteristics using estimates enables the interpretation of population dynamics and potential resource management^1^. In the results, the VBGF graphs for each sex of *O. punctatus* already showed significant differences from the 0 to 1 year age interval and the goodness of fit (R_n_) was above 0.2, indicating suitability. However, previous studies on *Portunus sanguinolentus* (Herbst, 1783) and *P. trituberculatus*, which belong to the same superfamily Portunoidea, did not show clear differences between the sexes in the VBGF graphs^54,62^. This can be easily explained by the growth performance index (φ'), which is a much more appropriate and robust method for comparing crustacean growth than simply comparing CW_∞_ and K values^59^. According to the results of Oh^54^, the growth performance indices of *P. trituberculatus* were 2.48 for males and 2.51 for females, whereas in this study, the values for *O. punctatus* were 4.10 for males and 3.87 for females, highlighting a stark contrast. These differences in crustacean growth may be due to environmental factors such as sea temperature^62^, indicating a need for future investigations into the correlations with marine environmental factors. Therefore, it can be concluded that not all species within the superfamily Portunoidea exhibit differences in growth curve patterns between sexes.

In Asian countries, ongoing overfishing of *O. punctatus* has led to a decrease in individual size, highlighting the need for institutional resource management policies based on ecological research findings^23^. Therefore, to protect the population of *O. punctatus* and enable sustainable fisheries, it is essential to establish resource management policies such as closed seasons and minimum size limits based on ecological data. Future reproductive ecological studies combined with the sex-specific growth characteristics estimated in this study can provide strengthened data to serve as a basis for policy formulation.

## Data Availability

The data generated during and/or analyzed in the current study are available from the corresponding author upon reasonable request.
